# The Use of Dehydrated Amniotic Membrane Allograft for the Augmentation of Dural Repair in Craniotomies

**DOI:** 10.7759/cureus.2586

**Published:** 2018-05-07

**Authors:** Daniel G Eichberg, Sheikh C Ali, Simon S Buttrick, Ricardo J Komotar

**Affiliations:** 1 Neurological Surgery, University of Miami Miller School of Medicine, Miami, USA; 2 College of Osteopathic Medicine, Nova Southeastern University, Fort Lauderdale, USA; 3 Neurological Surgery, University of Miami Miller School of Medicine , Miami, USA

**Keywords:** brain tumor, cerebrospinal fluid leak, cerebrospinal fluid leak, craniotomy, dehydrated amniotic membrane, dural repair, neurosurgery

## Abstract

Background: In cranial neurosurgery, primary watertight dural closure is the standard method of post-craniotomy dural repair. However, cerebrospinal fluid (CSF) leaks, pseudomeningoceles, postoperative infections, and dural scarring are possible complications, even when a meticulous technique is implemented. For this reason, materials that enhance the dura’s ability to create a watertight seal, inhibit the inflammatory response, and prevent disease transmission are sought. Dehydrated amniotic membrane (DAM) allograft appears to facilitate these properties, as studies have shown that it improves wound healing, prevents scar tissue formation, promotes epithelialization, and inhibits bacterial growth. We detail the use of a DAM allograft to augment dural closures for craniotomies.

Methods: We conducted a pilot study, retrospectively reviewing our institution’s database of craniotomies that utilized DAM to supplement dural closure.

Results: A total of 122 cases, including 18 initial craniotomies for infratentorial lesions, 102 initial craniotomies for supratentorial lesions, one re-do craniotomy for supratentorial recurrent glioma, and one craniotomy for an anterior skull base schwannoma used a DAM allograft to augment dural closure. Only one complication occurred (0.8% complication rate), which was a superficial wound infection requiring washout without craniectomy. No CSF leaks occurred.

Conclusions: This pilot study demonstrates that dehydrated amniotic membrane allograft can be safely utilized as an adjunct during dural closures for craniotomies.

## Introduction

After craniotomy with a dural opening, sutured watertight dural repair is the preferred method of closure, as it is believed to minimize the risk of cerebrospinal fluid (CSF) leakage and infection. However, tissue loss or dural compromise can make this technique unfeasible, especially in cases of prior radiation or revision craniotomy. Further, even when the ideal dural closure technique is employed, postoperative complications, such as CSF leaks, meningitis, abscesses, pseudomeningoceles, and dural scarring, may occur, possibly due to holes made in the dura by the suture needle [1].

When a primary dural closure is not possible, neurosurgeons must repair the dura with a dural reinforcement material or substitute, such as an allograft, xenograft, or synthetic biomaterial, each with their respective drawbacks [2]. Autologous tissues, such as pericranium, temporal fascia, and fascia lata, avoid the risk of disease transmission and an immunologic rejection. However, harvesting these tissues increases surgical invasiveness, which may increase the overall risk of local tissue damage, infection, or pain. Dural allografts, such as lyophilized cadaveric human dura mater, are not routinely used in cranial neurosurgery since cases have been reported demonstrating the transmission of prion diseases, such as Creutzfeldt-Jakob disease [2-4]. Synthetic biomaterials have largely been abandoned due to the excessive generation of an inflammatory reaction resulting in underlying brain irritation, meningitis, hemorrhage, graft encapsulation, and inordinate scar formation [5-7]. Thus, there is great demand for a dural augmentation material that does not generate an inflammatory or fibrotic scar response, creates a watertight seal, and does not transmit disease.

Dehydrated amniotic membrane (DAM) has been shown to inhibit fibrosis and scar formation, prevent an immune response, promote epithelization, and have antibacterial and anti-inflammatory properties [8]; thus, it may be an ideal dural substitute. DAM has been successfully utilized in wound closure in multiple surgical subspecialties, including plastic surgery [9-10], general surgery [11], otolaryngology [12], maxillofacial surgery [13], ophthalmology [14], and spinal surgery [15-16]. However, its use is not described in the cranial neurosurgery literature. As such, its efficacy and safety profile in cranial neurosurgery is unknown. In this pilot study, we present our institution’s experience using a DAM allograft during dural closure for craniotomies in 122 patients.

## Materials and methods

Patient selection

All patients undergoing primary, revision, or re-do craniotomy at our institution from August 2016 to February 2017 underwent dural closure supplemented by Cygnus Solo dehydrated amniotic membrane (Vivex Biomedical, Miami, Florida, US) and were included in the current retrospective study (Table 1). Cygnus Solo is United States Food and Drug Administration (FDA) approved under the criteria of minimal manipulation and homologous use as a wound covering or barrier for dura mater defects.

**Table 1:** Summary of patients' demographic characteristics

Mean age, years (range)		58 (19-91)
Gender (% of cohort)		45 (36.9%) male, 77 (63.1%) female
Procedure (% of cohort)	Initial infratentorial craniotomy	18 (14.8%)
	Initial supratentorial craniotomy	102 (83.6%)
	Revision supratentorial craniotomy	1 (0.8%)
	Craniotomy for anterior fossa skull base lesion	1 (0.8%)
Pathology (% of cohort)	Metastasis	34 (27.9%)
	Meningioma	31 (25.4%)
	Glioblastoma multiforme	24 (19.7%)
	Low-grade glioma	11 (9.0%)
	High-grade glioma	5 (4.1%)
	Colloid cyst	3 (2.5%)
	Hemangioblastoma	2 (1.6%)
	Encephalitis	2 (1.6%)
	Aneurysmal bone cyst	1 (0.8%)
	Anterior skull base schwannoma	1 (0.8%)
	Cavernous malformation	1 (0.8%)
	Ependymoma	1 (0.8%)
	Epidermoid cyst	1 (0.8%)
	Pineal parenchymal tumor of intermediate differentiation	1 (0.8%)
	Hamartoma	1 (0.8%)
	Hemorrhagic lesion of nondiagnostic pathology	1 (0.8%)
	Lymphoma	1 (0.8%)
	Recurrent glioma	1 (0.8%)

The senior neurosurgeon’s (RJK) clinical database was searched to obtain all relevant patients, and the electronic medical record was analyzed to determine patient demographic data, operative information, and post-operative course. Institutional review board (IRB) approval was obtained before proceeding with clinical database review (IRB # 20170675), along with a waiver of informed consent.

DAM layering technique

Watertight dural closure using running 4-0 braided nylon suture is attempted in all cases. If this cannot be achieved, the dura is approximated with interrupted sutures to create a scaffold for the dural substitute. Next, a layer of DAM is placed over the dura, epithelial side up. Afterward, a sheet of a bovine collagen dural substitute (Duragen, Integra LifeSciences, Plainsboro, New Jersey, US) is placed over the DAM. Finally, the bone flap is secured in place with titanium plates and screws, followed by skin closure with sutures.

## Results

A total of 122 cases (Table 1) using DAM allografts was performed at our institution from August 2016 to February 2017 by the senior neurosurgeon (RJK). Table 1 shows a summary of patient demographics. The average patient age was 58.0 years. Forty-five patients (36.9%) were male and 77 (63.1%) were female.

Eighteen (14.8%) initial craniotomies for infratentorial lesions, 102 (83.6%) initial craniotomies for supratentorial lesions, one (0.8%) revision craniotomy for a supratentorial recurrent glioma, and one (0.8%) initial supratentorial craniotomy for an anterior skull base schwannoma used a DAM allograft to supplement dural closure. The three most common pathologies included metastases, meningiomas, and glioblastoma multiforme.

One postoperative complication occurred, which consisted of a superficial wound infection requiring washout without craniectomy (Table 2). No cases were complicated by a CSF leak. No revision craniotomies were complicated by a postoperative wound infection or CSF leak.

**Table 2:** Complication rates Abbreviation: CSF: cerebrospinal fluid

	Number of cases (%)	Wound infections (%)	CSF leaks (%)
Initial craniotomy for infratentorial lesion	18 (14.8%)	1 (1.0%)	0
Initial craniotomy for supratentorial lesion (non-skull base)	102 (83.6%)	0	0
Revision craniotomy for supratentorial lesion (non-skull base)	1 (0.8%)	0	0
Craniotomy for anterior skull base schwannoma	1 (0.8%)	0	0
All cases	122 (100%)	1 (0.8%)	0

After bone flap removal in the case of the revision craniotomy for recurrent glioma, the DAM previously placed during the closure of the original craniotomy for tumor resection was seen intact and fully integrated into the surrounding dura.

## Discussion

The standard technique for dural repair after the completion of craniotomy is a sutured, watertight closure. However, watertight closure is not always feasible, especially for convexity meningiomas where the dura has been purposefully removed, and in cases of prior surgery or prior radiation exposure, in which the dural integrity is compromised. Additionally, even when a watertight dural closure is achieved and confirmed by an intraoperative Valsalva maneuver, postoperative CSF leak, pseudomeningocele, infection, and dural scarring may occur [1]. Therefore, a dural augmentation material that reduces the rates of these complications is desirable.

Ideally, a dural supplementation material should promote epithelization to integrate with the native dura but not allow for adhesions to form to the underlying brain. Additionally, it should be immunologically inert to minimize the risk of inflammation, scarring, and immunologic rejection. Finally, the optimal dural adjunct is sterile to minimize the risk of pathogenic transmission and non-toxic to avoid local tissue damage [2]. A dehydrated amniotic membrane (DAM) may confer all of these characteristics.

Because it lacks numerous cell surface antigens that generate an immunologic response, DAM is considered to be a non-immunogenic material and, therefore, does not trigger a graft rejection reaction [17-18]. Amniotic epithelial cells have also been demonstrated to have anti-inflammatory properties [8,19]. Additionally, amniotic tissue has been shown to inhibit bacterial growth and have antimicrobial properties [20-22].

Further, DAM is known to reduce fibrosis due to the presence of numerous growth factors, which are responsible for the proliferation, migration, and differentiation of epithelial cells and epithelialization. For this reason, fetuses have been shown to heal wounds without forming scars [23]. This anti-scar formation property has popularized DAM in burn repair and plastic surgery applications [9-10]. If a revision surgery is needed, postoperative dural scar formation from the initial surgery may complicate dural reopening if the dura is adherent to the brain due to scar formation. Additionally, prior scar formation may complicate the re-closure of the dura, potentially increasing the risk of post-operative infection and CSF leaks. For these reasons, the postoperative risk of dural scar formation is ideally minimized. Our experience with DAM appears to be consistent with the reported findings of decreased postoperative scar formation. In the case of a re-operative craniotomy for a recurrent glioma, we report that DAM previously placed during the original craniotomy was found intact and fully integrated into the surrounding dura, with minimal fibrosis or scar tissue.

Successful DAM use has been described in numerous non-neurosurgical clinical applications, such as corneal repair, burn treatment, and oral cavity reconstruction [8-13]. In neurosurgery, autologously harvested amniotic membrane has been used to repair dural defects in myelomeningocele [16].

Although the human cranial neurosurgery applications of DAM have not been thoroughly investigated, an *in vivo* rat cranial surgery model demonstrated that human xenograft DAM was efficacious and had an adequate safety profile. Tomita et al. (2012) developed a rat model in which 20 rats underwent a skull base craniotomy with the excision of dura and the placement of dehydrated human amniotic membrane [2]. None of the cases experienced clinical adverse reactions related to the DAM or CSF leak. Interestingly, on a histological examination at six months postoperatively, the dehydrated human amniotic membrane was completely resorbed and replaced with a rat-derived membranous structure.

Published infection rates for craniotomies vary widely; a recent matched case-control study of 2819 patients described a postoperative infection rate of 2.47% [24]. Our series’ postoperative infection rate of 0.8% is comparable to that of reported infection rates, suggesting that DAM does not increase the postoperative infection rate. Similarly, the reported rates of postoperative CSF leaks differ among studies and craniotomy locations; leaks may complicate between 4% and 17% of craniotomies for posterior fossa lesions [25]. In our series of 122 craniotomies, including 18 craniotomies for posterior fossa lesions, none were complicated by postoperative CSF leaks. These results suggest that DAM does not contribute to the increased risk of CSF leaks.

Because re-operative craniotomies are considered a higher risk for a postoperative CSF leak or infection, the finding that our reported case of a re-operative craniotomy for a recurrent tumor in our series had no postoperative complications provides further evidence to the claim that DAM is a promising dural supplement for the closure of craniotomies.

Our pilot study is limited by the fact that it has no control group that used closure without DAM or with other dural adjuncts, and it was not randomized. While our reported infection rates and CSF leak rates were comparable to or lower than previously reported rates [24-25] because the study was not randomized, the findings could be biased due to confounding variables. Further, the interpretation of the data is complicated by the fact that the patients in our study received a sheet of bovine collagen dural substitute layered on top of the DAM; thus, the outcomes may be due to both materials. Future studies with patients randomized into groups with other dural closure techniques are required to validate our study.

While this retrospective pilot study does not prove the superiority of DAM over other dural adjuncts, or the efficacy of use, we demonstrate that it has an adequate safety profile with no complications directly related to its use in closures for craniotomies. We also report very low CSF leak rates and infection rates, particularly in craniotomies for infratentorial lesions. Future randomized controlled studies are warranted to determine the safety profile and efficacy in comparison to other closure techniques and dural adjuncts.

## Conclusions

We report the use of a dehydrated amniotic membrane (DAM) allograft during the closure of craniotomies. With this pilot study, we report an adequate safety profile with no adverse reactions directly related to the DAM product. Future studies are warranted that compare DAM with other closure techniques and dural supplements to prove efficacy.
